# Paternal heat exposure causes DNA methylation and gene expression changes of *Stat3* in Wild guinea pig sons

**DOI:** 10.1002/ece3.1993

**Published:** 2016-02-28

**Authors:** Alexandra Weyrich, Stephanie Benz, Stephan Karl, Marie Jeschek, Katarina Jewgenow, Joerns Fickel

**Affiliations:** ^1^ Leibniz‐Institute for Zoo and Wildlife Research (IZW)Alfred‐Kowalke‐Str. 17D‐10315BerlinGermany; ^2^ Berlin Center for Genomics in Biodiversity ResearchKoenigin‐Luise‐Str. 6‐814195BerlinGermany; ^3^ Potsdam UniversityKarl‐Liebknecht‐Str. 22‐2414476PotsdamGermany

**Keywords:** Adaptation, DNA methylation, nonmodel species, Paternal effects, thermoregulation, transgenerational epigenetic inheritance

## Abstract

Epigenetic mechanisms convey environmental information through generations and can regulate gene expression. Epigenetic studies in wild mammals are rare, but enable understanding adaptation processes as they may occur in nature. In most wild mammal species, males are the dispersing sex and thus often have to cope with differing habitats and thermal changes more rapidly than the often philopatric females. As temperature is a major environmental selection factor, we investigated whether genetically heterogeneous Wild guinea pig (*Cavia aperea*) males adapt epigenetically to an increase in temperature, whether that response will be transmitted to the next generation(s), and whether it regulates mRNA expression. Five (F0) adult male guinea pigs were exposed to an increased ambient temperature for 2 months, corresponding to the duration of the species' spermatogenesis. To study the effect of heat, we focused on the main thermoregulatory organ, the liver. We analyzed CpG‐methylation changes of male offspring (F1) sired before and after the fathers' heat treatment (as has recently been described in Weyrich et al. [*Mol. Ecol*., 2015]). Transcription analysis was performed for the three genes with the highest number of differentially methylated changes detected: the thermoregulation gene Signal Transducer and Activator of Transcription 3 (*Stat3*), the proteolytic peptidase gene *Cathepsin Z* (*Ctsz*), and *Sirtuin 6* (*Sirt6*) with function in epigenetic regulation. *Stat3* gene expression was significantly reduced (*P* < 0.05), which indicated a close link between CpG‐methylation and expression levels for this gene. The two other genes did not show gene expression changes. Our results indicate the presence of a paternal transgenerational epigenetic effect. Quick adaptation to climatic changes may become increasingly relevant for the survival of wildlife species as global temperatures are rising.

## Introduction

Past epigenetic studies have mainly focused on medical and developmental aspects in humans and model species, while studies on ecologically relevant traits in nonmodel species are still scarce (Richards 2006; Pertoldi and Bach 2007; Bossdorf et al. 2008). However, to cope with environmental factors, (wild) animals need to adapt to environmental changes, for example in habitat and temperature (Kilvitis et al. 2014). The regulation of gene expression is fundamental for immediate adaptation processes in the same generation. In addition, the inheritance of responses to experienced changes (adapted traits) is fundamental for long‐term adaptational memory and thus evolutionary processes. The mechanism regulating gene expression and conferring such immediate and inherited adaptation is “epigenetic response” (Jablonka and Raz 2009). This response is achieved by epigenetic marks, including DNA methylation, histone modifications, polycomb proteins, and siRNAs, which are controlling the accessibility of the chromatin to the transcriptional machinery. The best‐studied epigenetic modification is DNA methylation (e.g., Boyes and Bird 1991; Tate and Bird 1993; Jones and Takai 2001; Bird 2002).

In mammals, DNA methylation is mostly achieved by the covalent binding of a methyl (‐CH_3_) group to the 5‐carbon site of a cytosine, forming 5‐methylcytosine (5mC). It mainly occurs at single cytosine–phosphate–guanine dinucleotides (CpG) of which about 80% are methylated (mCpG). CpG methylation occurring in the promoter region of a gene is often strongly associated with gene repression (He et al. 2000; Deaton and Bird 2011). Thus, this epigenetic mechanism can regulate the gene activity, without changing the DNA sequence underneath. The most intriguing aspect of this mechanism is that the epigenetic pattern is dynamically changing in response to a changing environment in the same generation (“immediate response” or “epigenetic plasticity”) and can also be transmitted to the next generation (“inherited response” or “transgenerational epigenetic plasticity”). Although the exact mechanisms of transgenerational epigenetic inheritance and the differences between maternal and paternal effects are still unclear, the phenomenon itself is progressively confirmed (Weaver et al. 2004; Champagne 2008; Carone et al. 2010; Ng et al. 2010).

To ensure epigenetic plasticity through generations, DNA methylation patterns must either survive epigenetic reprogramming during early embryogenesis (Haaf 2006) or must be re‐established afterward. Such “survivors” are parentally imprinted genes that are important in early embryonic development (Reik et al. 2001). However, recent studies showed that nonimprinted genes may also “survive” the reprogramming process (Borgel et al. 2010; Wei et al. 2014). Due to the close intrauterine relationship, as well as maternal care after birth, maternal effects on the offspring are more evident than paternal effects. As a result, researchers mainly focused on maternal effects and transmission (e.g., Weaver et al. 2004; Wolff et al. 1998), whereas paternal effects have been widely neglected (e.g., Carone et al. 2010; Dias and Ressler 2014). An advantage of examining paternal effects is that effects during pregnancies and behaviorally induced effects can be excluded (in many mammal species, males are not actively involved in rearing the offspring), simplifying detection of molecular mechanisms (Curley et al. 2011). Thus, to achieve a comprehensive understanding of transgenerational epigenetic effects, paternal effects need to be studied too, as well as their role in adaptation.

Besides, studying paternal effects in wild species is especially crucial, because in most wild mammal species, males are the dispersing sex and thus have to cope with more rapid changes in habitats and temperature, while females are often philopatric. This also holds true for the Wild guinea pig, *Cavia aperea*, which is living in harem structures with one dominant male, who defends several females from other males (Asher et al. 2008). The nondominant, roaming males thereby need to adapt quickly to new habitats and temperatures before finding accepting female(s). Climate changes have always impacted on the evolution of species, and ambient temperature is one major external selection factor animals have to cope with. The species long‐term survival depends on their short‐term responses. The ability to convey heritable phenotypic plasticity to descendant generations will likely contribute to fitness increase and thus to improved chances of survival.

We here investigated the potential paternal contribution to an epigenetically adaptational response to increased ambient temperature in a wild species. Therefore, as described in detail in a recent publication (Weyrich et al. 2015), we temporally exposed adult male Wild guinea pigs to an increased ambient temperature and let them mate to the same females before and after the heat exposure. We then analyzed whether their sons – one group sired before, the other after the fathers' heat treatment – showed altered DNA methylation patterns compared with the situation prior to the heat exposure (experimental setup in Fig. S1). To study the epigenetic responses to heat, we here focused on the main metabolic, heat‐producing and thus thermoregulatory organ, the liver. Mammals, such as *C. aperea*, are homeostatic; that is, they compensate external thermal fluctuations (thermoregulation) to maintain internal temperature (homeostasis). In endotherms, thermoregulation is a crucial and complex process to ensure optimal energy usage at a given temperature (Sonna et al. 2002; Boyles et al. 2011). It is regulated by many genes of which the Signal Transducer and Activator of Transcription 3 gene (*Stat3*) (also named: Acute Phase Response Factor) is crucial for maintaining temperature homeostasis (Sonna et al. 2002). *Stat3* becomes activated by various cytokines and regulates – depending on the tissue type – the expression of genes essential for systemic thermoregulation, embryogenesis, and immune response (Durant et al. 2010; Qi and Yang 2014). Due to its role in many different physiological processes, *Stat3* is an important target for investigation.

In this study, we aimed to examine whether heat exposure of fathers would affect the methylation patterns in their F1 sons (measured in comparison with F1 sons sired either before (F1_C_) or after paternal heat exposure (F1_H_)) and whether these altered patterns would influence gene expression. With our approach, we address both (1) the paternal contribution of epigenetic transmission and (2) understanding the epigenetic adaptability in the context of globally rising temperatures. Both may contribute to a fitness increase of the offspring and would thus play an important role in evolution.

## Materials and Methods

### Animal care and treatment

All husbandry and experimental procedures were approved of by the German Committee of Animal Welfare in Research (permit no. V3‐2347‐35‐2011). Wild guinea pigs originating from Argentina and Uruguay (Asher et al. 2008) were obtained from F. Trillmich (University of Bielefeld) and housed at the IZW field station in Niederfinow, Germany. Wild guinea pigs are polyoestric. Male cavies were born in mid‐November 2010 and females in April–May 2011. Mating took place in January/February 2012 [control] and in September 2012 [heat]. Animals were housed in short‐tunnel‐connected indoor‐outdoor‐enclosures (1.3 m^2^) under natural photoperiod and temperature conditions. All animals were fed guinea pig pellets (Altromin Spezialfutter GmbH & Co. KG, Lage, Germany). Water and hay were provided ad libitum*,* and supplementary apples, peppers, or carrots were given daily. Vitamin C was added to the drinking water once a week. During a two‐month period, the indoor cages (80 × 80 cm^2^) were placed on a heating plate (Candor GmbH Leipzig) which heated the floor to a temperature of 30°C (covering a full cycle of spermatogenesis) (Fig. S1). The slightly cooler edges of the heating plate were fenced off by a mesh, installed in 5 cm distance from the cage wall, reducing cage size to 70 × 70 cm^2^. The experiment to assess the influence of environmental stressors was carried out with five adult males kept exclusively indoors (60 days, 24 h; 30°C).

Each of the five males mated with two females before (control) and after heat exposure (heat) by introducing them to the female's cage. After an observed mating, males were transferred back to their own cage. Males were mated with the same two females before and after heat exposure. Mating resulted in 16 F1 sons from the control mating (F1_C_ *N* = 16) and 18 from the mating after heat exposure (F1_H_ *N* = 18). Seven days after birth, whole livers (the main thermoregulatory and metabolic organ) of F1 sons were harvested and individually sequenced (*N* = 34).

### Reduced representation bisulfite sequencing

We performed Reduced Representation Bisulfite Sequencing (RRBS) (Meissner et al. 2005) to profile global methylation changes among heat and control groups. RRBS was performed, and data were analyzed as previously described (Weyrich et al. 2015). Reads sequenced on a HiSeq 2000 (Illumina) were mapped against an in‐house‐generated *C. aperea* reference sequence (http://www.ncbi.nlm.nih.gov/biosample/2252454; Acc.No. AVPZ00000001‐AVPZ00003131, Weyrich et al. 2014) using bismark mapper (Krueger and Andrews 2011).

### DNA methylation level analysis and group comparison

The bisulfite conversion rate was calculated as the number of mapped nonmethylated CpGs divided by the total number of mapped CpGs. After bisulfite treatment and alignment to the reference sequence, Cs in a read that mapped to a C in the reference were assumed to have been methylated cytosines (mCs). Thymines that mapped to a C position were regarded as formerly unmethylated cytosines (Cs), respectively (converted to U by bisulfite and substituted by subsequent PCR). Accordingly, the methylation level of each cytosine position was calculated as the number of reads mapping to this position and carrying a C divided by the number of reads carrying either C or T at this position.

Methylation level was calculated by the equation:

$$\frac{C}{C+T}=\text{methylation level per specific mC site}$$

We compared methylation states of sons before (control) and after the fathers' heat treatment. Sites with coverage of ≤5 per sample per group were excluded from the analysis. Significance of differences was determined using Fisher's exact test. Strong hyper‐ and strong hypomethylation were defined as absolute methylation difference greater than 30% (Gu et al. 2010).

### RNA extraction and cDNA synthesis

RNA was extracted from 20 mg of liver tissue of the sacrificed guinea pigs using the innuSPEED Tissue RNA kit (Analytik Jena, Germany) and peqGOLD DNase I Digest Kit (Peqlab, Erlangen, Germany). RNA extracts (elution with 50 *μ*L H_2_O) were stored at −80°C. RIN was determined as an objective indication of RNA quality (Schroeder et al. 2006) using the Agilent 2100 bioanalyzer and a RNA 6000 Pico Kit following the manufacturer's guidelines. RIN values for all samples were between 5.9 and 8.3 (SD = ±0.7). RNA samples were diluted to a final concentration of 25 ng/*μ*L in a volume of 22 *μ*L. The Revert Aid First Strand cDNA Synthesis Kit (Thermo Scientific, Munich, Germany) and its associated protocol were applied, including 100 mM Oligo(dT)_18_. The obtained cDNA was frozen and stored at −20°C.

### Primer design and verification

Primers (Table 1) were designed based on the *C. aperea* whole genome sequence (Weyrich et al. 2014) using the Geneious® software. For maximum quantification accuracy, primers producing amplicons of 120–200 bp were chosen with an optimal annealing temperature (*T*_a_) of 55°C and a length of 20+/− 2 nucleotides. Theoretical *T*_a_ values were calculated with the CFX Manager Software (Bio‐Rad GmbH, Munich, Germany). The cDNA template for PCR testing was freshly synthesized from randomly selected samples. Negative controls were set up for each primer pair. A positive PCR control was prepared using the *Stat3* primer pair. For visualization of PCR products, a 1.5% agarose gel was prepared (1.5 g agarose GOLD^®^ (Peqlab) + 100 mL 1x TBE buffer, 3 *μ*L GelRed^™^ (Biotium, Cologne, Germany)). 5 *μ*L PCR product was added to 1 *μ*L loading dye (6x orange DNA loading Dye; Fermentas, St. Leon‐Rot, Germany). The 50‐bp ladder (O'GeneRuler^™^ 0.1 *μ*g/*μ*L, Thermo Scientific) was used for size determination. PCR reaction conditions for primer pairs were optimized. Amplicons were verified in size using agarose gel electrophoresis as well as by Sanger sequencing (Sanger et al. 1977) using the BigDye^®^ Terminator v3.1 Cycle Sequencing Kit and BigDye^®^ XTerminator^™^ Purification Kit (Life Technologies, Darmstadt, Germany). Capillary electrophoresis was performed using the ABI Prism 3130xl Genetic Analyzer and associated Collection software v. 3.0 (Life Technologies). Sequences were analyzed using the Sequence Analyser^®^ v. 5.2 (Life Technologies) and BioEdit v. 7.2.5 software (Hall 1999). NCBI BLAST search finally confirmed sequence specificity.

**Table 1:** Gene primer sequences and annealing temperatures

Gene	Gene name	Sequence forward primer	Sequence reverse primer	*T*_a_ [°C]^a^
*Stat3*	Signal Transducer and Activator of Transcription 3	AAGTTCACATT|CTTGGGGTT	CTTCGAGACTGAGGTTTACC	50.2
*Ctsz*	Cathepsin Z	CTTGATGTTGATGCGATCTG	ATGAATACCTGACACCATCG	53.3
*Sirt6*	Sirtuin 6	GCATCAGTACTGCCTCAG	CTCGAAGGTGGTGTCGAA	52.5
*Hmbs*	Hydroxymethylbilane synthase	CTTGACACTGCACTGTCCAAGAT	GCAGATGGCTCCAAT GGTG	58.8
*B2 m*	Beta‐2‐microglobulin	CAAGTGTATTCCCGTCACCC	GTCTGACATCTCCACATTGTCTATC	55.9
*Gapdh*	Glyceraldehyde‐3‐phosphate dehydrogenase	CTCACTTGAAGGGTGGTGC	CAACCGACACATTAGGTGTG	55

a *T*_a_, annealing temperature.

### Real‐time quantitative PCR

Quantitative PCR experiments were performed on the CFX96 Real‐Time System C1000 Thermal Cycler equipped with CFX Manager software (both Bio‐Rad). As standard curve, a 1:6 mixture of all 34 samples with five serial dilutions (in steps of 1:10) allowed calculation of run efficiency (E). Samples, standards, and controls were run in duplicates and prepared using the SsoFast EvaGreen^™^ Supermix on white optical plates. All qPCR runs were prepared by the same analyst and performed on the same platform to minimize inter‐run variation, thus minimizing distortion of results (Derveaux et al. 2010).

### Real‐time quantitative PCR data analysis

Run quality was assessed based on the efficiency (*E*) and *r* ^2^ values generated by the software. *E*‐values > 90% and *r* ^2 ^ > 0.98 (no between‐cycle variation in efficiency) were accepted. If the standard deviation between duplicates was > 0.4, samples were repeated. Efficiency‐corrected expression values (*Q*) (Pfaffl et al. 2002) were calculated from the software‐generated cycle threshold values (Ct) according to the equation: *Q* = *E* ^−Ct^

Relative quantification relies on the normalization of the expression values for the target genes with reference genes, accounting for intraindividual and technical variation. Reliable normalization requires the use of several reference genes showing stable expression for the tested tissue and treatment. Normalization of the *Stat3* expression values was performed using the software GeNorm to define a combination of three reference genes resulting in the most stable expression levels (Weyrich et al. 2010). Eventually, we used *Hmbs* (efficiency *E* = 110.4%), *B2 m* (*E* = 108.8%), and *Gapdh* (*E* = 90%) (Table 1). For each sample, a normalization factor (NF) was calculated based on the geometric mean (GM) of the Q values for the three reference genes (Qr) (Vandesompele et al. 2002): $\text{NF}1=\text{GM}=\sqrt{\text{Qr}1\times \text{Qr}2\times \text{Qr}3}$

Normalization factor was then applied to expression values for the target genes using the efficiency‐corrected ΔCt method (Pfaffl et al. 2002), giving the normalized expression values (NE) used for statistical analysis:

$$\text{NE}1=\frac{Q1}{\text{NF}1}$$

With *Q*1 = efficiency‐corrected expression value for target gene 1, NF1 = normalization factor for sample 1.

### Statistical analysis

We applied Fisher's exact test for methylation levels and a paired Wilcoxon signed‐rank test to test for significant differences between F1_C_ and F1_H_ for normalized expression values using R v.3.1.1 (http://cran.r-project.org/bin/windows/base/). A paired Wilcoxon signed‐rank test was also applied to investigate a potential maternal effect; wherefore, we first averaged the expression values of the sons per father–mother combination (*n* = 10). The plots were generated in R with ggplot2 v1.0.0 and are displayed as whisker plots with 1.5*IQR (http://cran.r-project.org/web/packages/ggplot2/citation.html). Normalized expression values were also tested for significant associations with specific methylation changes using Pearson's test in R.

## Results

### Methylation changes

**Figure 1:**
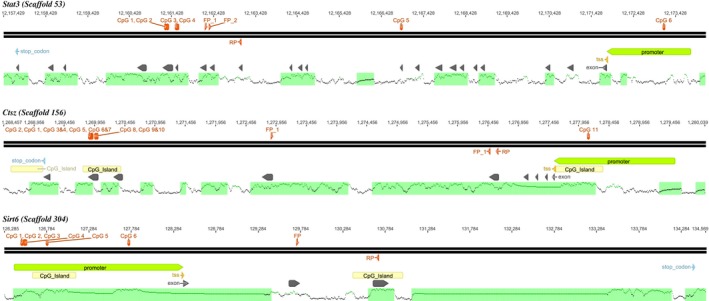
Gene maps. For *Stat3*,* Sirt6* and *Ctsz* CpG sites which were significantly changed in their methylation are displayed (red dots) at their specific genomic positions (numbers in the upper line) and in context of annotated region: promoter region (green arrow), exons (gray arrows), transcription start side (TSS, yellow arrow), stop codon (blue arrow), CpG islands (yellow arrows, Takai and Jones 2002) and the lower section contained CGIs (black lines) which were calculated by the percentage of CG dinucleotides, depicted as frequency graph. QPCRs are marked: forward primer (FP) and reverse primer (RP). For *Stat3* and *Sirt6,* cDNA binding was ensured using an exon‐spanning forward primer indicated by FP_1 and FP_2 (Figures were generated using Geneious^®^ v. 8.0.4.).

After comparing CpG‐methylation levels of the control group with those of the heat group (F1_C_ vs. F1_H_), we detected significant differences in several genomic loci (*P* < 0.05; Fisher's exact test), indicating paternal epigenetic transgenerational inheritance. We then focussed on the three genes with the greatest abundance of significant mCpG‐differences (occurring in promoters as well as in coding sequences) between control and heat group: the thermoregulatory gene, *Signal Transducer and Activator of Transcription 3* (*Stat3);* the *Cathepsin Z* gene (*Ctsz*), a lysosomal cysteine proteinase with function in tumorigenesis; and the *Sirtuin 6* gene (*Sirt6*), a gene with function in intercellular and epigenetic regulation (Fig. 1). We investigated methylation levels for each CpG in F1_C_ (*N* = 16) and F1_H_ sons (*N* = 18) (Table S1).

**Figure 2:**
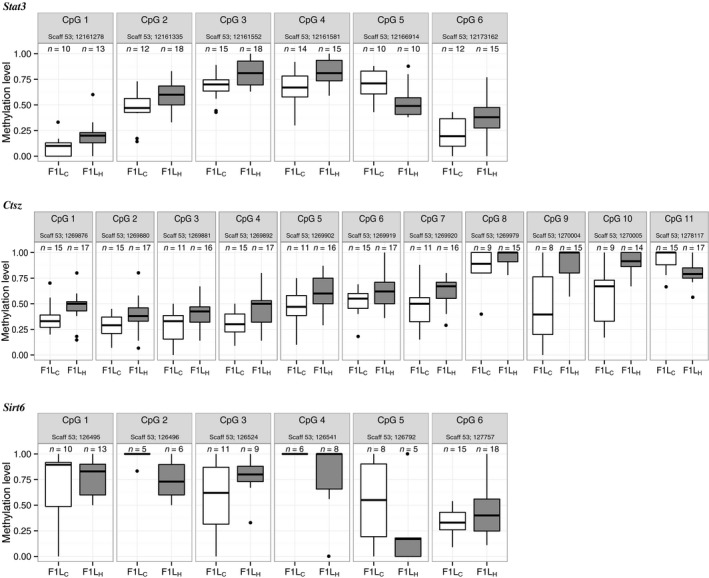
CpG‐specific methylation levels. Boxplots show significant DNA methylation changes of the single CpG sites between F1_C_ (white boxes) and F1_H_ (gray boxes). Scaffolds (scaff) are indicated for the respective gene and genomic CpG positions for each CpG site. Only positions with a coverage ≥ 5 in the respective individual at the certain CpG position are incorporated (*n* = x). *Stat3* is generally higher methylated in liver samples of F1_H_ compared with F1_C_ in all CpGs but CpG 5. Those results are similar to those of *Ctsz* which shows lower methylation in F1_H_ only in CpG 11. In contrast, methylation levels of *Sirt6* varied more frequently in F1_H_ compared with F1_C_. Here, hypomethylation was detected at CpG 1, 2, 4, and 5 and hypermethylation at CpG 3 and CpG 6.

In the *Stat3* gene, six CpGs were differentially methylated, one in the promoter and five in the coding region (Figs. 1 and 2). While five of the six CpGs showed greater methylation levels in the F1_H_ group, one CpG had a higher methylation level in the F1_C_ group (Fig. 2).

Similar to the *Stat3* methylation pattern, in *Ctsz,* hypermethylation of CpGs was also mostly detected in the F1_H_ group (in 10 of the 11 CpGs). Heat exposure of fathers led to the hypomethylation of CpG 11 in F1_H_. Interestingly, this CpG was the only one located in the promoter region. In *Sirt6,* all differentially methylated CpGs were located in the gene's promoter region. Four of these CpGs were hypomethylated and two were hypermethylated in F1_H_ individuals relative to control individuals, reflecting varying levels of methylation.

### Gene expression analysis

**Figure 3:**
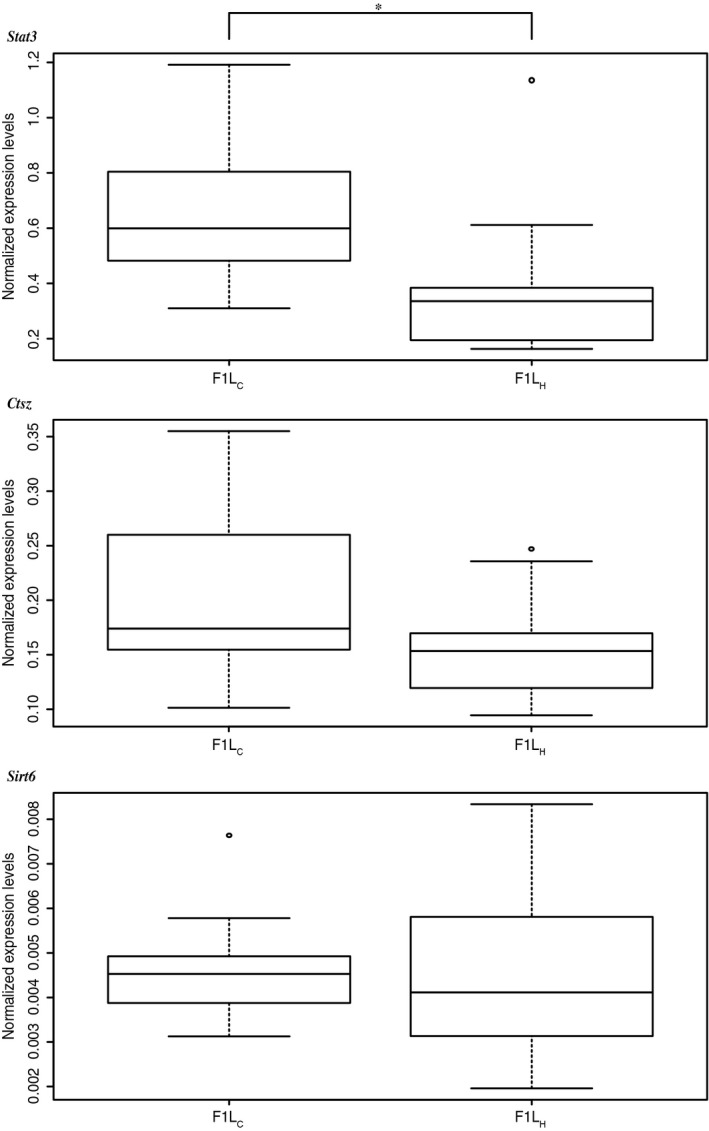
Expression levels. Boxplot of *Stat3, Sirt6,* and *Ctsz* normalized expression levels of control F1_C_ and heat F1_H_ group. *Stat3* showed a significant twofold change between groups (*P = 0.00069). In comparison with *Stat3* and *Ctsz,* the mRNA expression values of *Sirt6* were rather low.

Measuring expression levels in both F1_C_ and F1_H_ using quantitative PCR (qPCR) revealed significant differences in gene expression of *Stat3*, but not of *Sirt6* or *Ctsz* (Fig. 3; Table S1). *Stat3* mRNA levels were significantly lower in F1_H_ than in F1_C_ (mean F1_C _= 0.635, SD = ±0.233; mean F1_H _= 0.376, SD = ±0.289; paired Wilcoxon singed‐rank test *P* = 0.01099). By applying a paired Wilcoxon signed‐rank test to the averaged expression values of the sons per father–mother combination (*n* = 10), we verified treatment specificity and excluded maternal effects on gene expression (*P* = 0.03125). These results showed that DNA methylation in F1 sons was paternally influenced, indicating transgenerational epigenetic plasticity.

### Linking *Stat3* DNA methylation and gene expression

Focussing on *Stat3*, we applied a Pearson correlation to investigate the association between paired mRNA expression values and the methylation levels at each of the six single CpGs (Figs. 1 and 2, Table 2). There were clear, yet weak associations between methylation differences at each of the tested CpG positions (CpG 1 to CpG 6) and gene expression changes. Differential methylation at position 1 and 3 had the strongest (negative) association with gene expression (*r*_1 _= −0.371 and *r*_3 _= −0.418), followed by CpG 4 and CpG 6 (*r*_4 _= −0.167, *r*_6 _= −0.168).

**Table 2:** Correlation of methylation and expression levels of *Stat3*

	CpG 1	CpG 2	CpG 3	CpG 4	CpG 5	CpG 6^a^
Pearson's (*r*‐values)	−0.371	−0.141	−0.418	−0.273	0.019	−0.168

Pearson correlation coefficients (*r*‐values) showing associations of methylation levels and expression levels per CpG dinucleotide for *Stat3*.

a CpG located in promoter region.

[Correction added on 18 March 2016, after initial online publication on 28 February 2016. Citation and placement of tables 1 and 2 and reference to ‘Weyrich’ in the Abstract were incorrect and have been updated in this version.]

## Discussion

### Main findings

Our results demonstrate a paternal effect on DNA methylation patterns of the next generation after exposure to increased temperature. The investigation of three genes revealed that one of them *Stat3*, a key thermoregulation gene involved in numerous biological processes was not only differentially methylated between F1_C_ and F1_H_ siblings, but also significantly differentially expressed (*P* < 0.05). In F1_H,_ *Stat3* was hypermethylated in comparison with F1_C_ and gene activity reduced.

### Regulation of expression

Our findings add to the evidence (Ng et al. 2010; Wei et al. 2014) for paternally inherited changes in DNA methylation patterns and their impact on gene activity. Although the exact mechanism remains still unknown, the genomic location of CpGs at which methylation changes take place seems crucial, because promoter methylation has often been strongly associated with gene repression (He et al. 2000; Deaton and Bird 2011), while *intra*gene methylation can have either a gene silencing or an activation function (Hahn et al. 2011; Jjingo et al. 2012). Some studies describe one CpG/mCpG position as being sufficient for gene regulation (e.g., Ng et al. 2010), others suppose that stretches of differentially methylated CpGs, so‐called differentially methylated regions (DMRs), are the main regulatory units (Radford et al. 2014). Besides direct effects, DNA methylation can inhibit transcription also indirectly *via* a methyl‐binding (MBD) protein (Boyes and Bird 1991) and eventually interact with other epigenetic modifications, such as histone modifications and RNAi (Wei et al. 2014).

### *Stat3 –* Correlation results

In our study, *Stat3* differential CpG methylation for CpG 1–4 and CpG 6 was negatively associated with the gene's expression level (all hypermethylated in F1_H_) and positively associated (albeit weakly) for CpG 5 (hypermethylated in F1_C_), which was the only one located in a CG‐poor region (Fig. 2A). The strongest associations were detected for CpG 3 and CpG 1, both of which were located in the transcribed region of the gene. The site located in the promoter region, CpG 6, had only a weak negative correlation to gene expression. This was unexpected because methylation of mainly promoter regions had been shown to influence gene expression (Deaton and Bird 2011; He et al. 2011). However, in case of *Stat3,* the CpGs located in the transcribed region of the gene are likely candidates for having caused the expression differences observed. CpG methylation in transcribed regions is associated with both activation and deactivation of gene expression: in cells with low proliferation rates such as neural cells, it is linked to gene deactivation. In fast‐dividing cells such as liver cells, it is associated with gene activation and thus with higher expression levels (Hahn et al. 2011; Jjingo et al. 2012). The mechanism, by which intragenic methylation within the coding region increases a gene's activity, is currently poorly understood. It has often been suggested that it prevents uncontrolled transcription and promotes transcriptional elongation (Hellman and Chess 2007; Aran et al. 2011). It may also well be that the different CpGs act collectively, which would indicate a more complex interplay between the methylations/demethylations at different positions.

Correlations between specific DNA methylation changes and gene expression changes were weak. A reason might be the limited number of animals studied. However, despite the Wild guinea pig being a heterogeneous mammal species with phenotypic, genetic, epigenetic, and transcriptional variability and an average genetic dissimilarity among animals of ~0.17% (Weyrich et al. 2015), we found a significant response across individuals in the *Stat3* gene. This response across individuals of *Stat3* indicates an ecological relevance. It also identifies *Stat3* to be a suitable candidate for studies on globally changing temperatures.

The differentially methylated CpGs in the *Stat3* gene accompanied with gene expression changes (lower expression of *Stat3* in F1_H_ liver) suggest that the paternal experience transmitted via epigenetic transmission might contribute to a fitness increase of the offspring.

The gene product of *Stat3* itself is also interacting with epigenetic factors. In T cells, *STAT3* activates transcription of genes in co‐regulation with histone 3 lysine 4 trimethylation (H3K4me3), a marker with known transcription‐activating function (Durant et al. 2010).

We cannot fully exclude an age effect on DNA methylation as F0 males grew ~7.5 months older until the second mating after heat exposure. Studies on monozygotic human twins have shown that young twins (3 years) are indistinguishable in their DNA methylation patterns, whereas older twins (50 years) show substantial methylation changes (Fraga et al. 2005). Methylation patterns remain relatively constant for long periods, and those age‐related DNA methylation changes were observed after 15–20 years (Fraga et al. 2005, Fig. S5). Even when taking the shorter life span of the Wild guinea pigs into account, significant age‐related DNA methylation changes are not expected within a period of 7.5 months during adulthood.

### *Ctsz* and *Sirt6*

*Ctsz* and *Sirt6* were not affected in their gene activity, even though methylation had changed between F1_C_ and F1_H_. This was particularly surprising for *Sirt6* where all six differentially methylated CpGs were located in the promoter region.

However, recent studies have demonstrated that DNA methylation does not always correlate with gene expression (Huang et al. 2014), which thus indicates a more complex regulatory mechanism. We hypothesize that the lack of expression changes might reflect a regulatory mechanism, in which an increase in exposure time is positively correlated to an increase in the number of CpGs to be altered in their methylation in regulatory regions. Reaching this threshold of differentially methylated CpGs may alter gene expression (dosage effect). Following our hypothesis, in case of *Ctsz* and *Sirt6*, methylation changes were thus not strong enough to be of biological meaning. Changes might also occur later in life or in case the sons would again be exposed to temperature increase. Other reasons might be the age of the animals at the time of sampling. As the exact molecular and mechanistic basis for these observations remains largely unclear, further studies will be needed to address these questions.

### Comparison with other studies

As this is the first study examining the molecular effects of paternal heat stress on subsequent generations in a nonmodel mammalian species, comparisons with other studies in the field of paternal epigenetic transgenerational inheritance are limited. In rats, paternal high‐fat diet increased the risk of obesity and diabetes in female offspring (Ng et al. 2010), and low‐protein diet influenced hepatic gene expression and metabolic function in both female and male offspring (Carone et al. 2010). Behavioral conditioning in male mice changed gene expression and neurophysiology in male and female offspring (Moore et al. 2013).

### Evolutionary relevance

The evolutionary significance of epigenetic transgenerational inheritance of ecologically relevant traits is a subject of debate (Lachmann and Jablonka 1996; Takai and Jones 2002; Root and Schneider 2006; Moore et al. 2013). While the underlying mechanisms have not been fully understood, our results suggest that acquired responses to heat exposure are transmitted to the next generation in a nonmodel species. Its transmission to the next generation and this seemingly targeted response to heat is the prerequisite for epigenetically mediated microevolution. For a comprehensive assessment of the evolutionary significance of epigenetic transgenerational inheritance, long‐term studies including several subsequent generations will be needed.

### Outlook

Thermoregulation is of great ecological and evolutionary importance. Transgenerational epigenetic inheritance may be of great advantage to adapt to rapidly as well as persistently rising temperatures (Lachmann and Jablonka 1996; Root and Schneider 2006). In times of globally rising temperature, this mechanism might become even more important for species survival.

## Conflict of Interest

The authors declare no competing financial interests.

## Data and Materials Availability

Next‐generation sequencing data were uploaded to the National Center for Biotechnology Information Short Reads Archive (http://www.ncbi.nim.nih.gov/sra) and will be publicly accessible under the SRA study accession number SRP048942.

## Supporting information


**Fig. S1** Experimental set‐up.
**Table S1** Methylation and expression levels of *Stat3*.
