# Supplementary material for: Paternal heat exposure causes DNA methylation and gene expression changes of Stat3 in Wild guinea pig sons
Source: Ecol Evol. 2016 Feb 28;6(9):2657–66. doi: 10.1002/ece3.1993 (PMC4769883; doi:10.1002/ece3.1993)
Supplement: Supplementary file 1 — Fig. S1 Experimental set‐up. Table S1 Methylation and expression levels of Stat3. [file ECE3-6-2657-s001.docx]

**Supplementary data**

**Fig. S1 Experimental set-up**

**
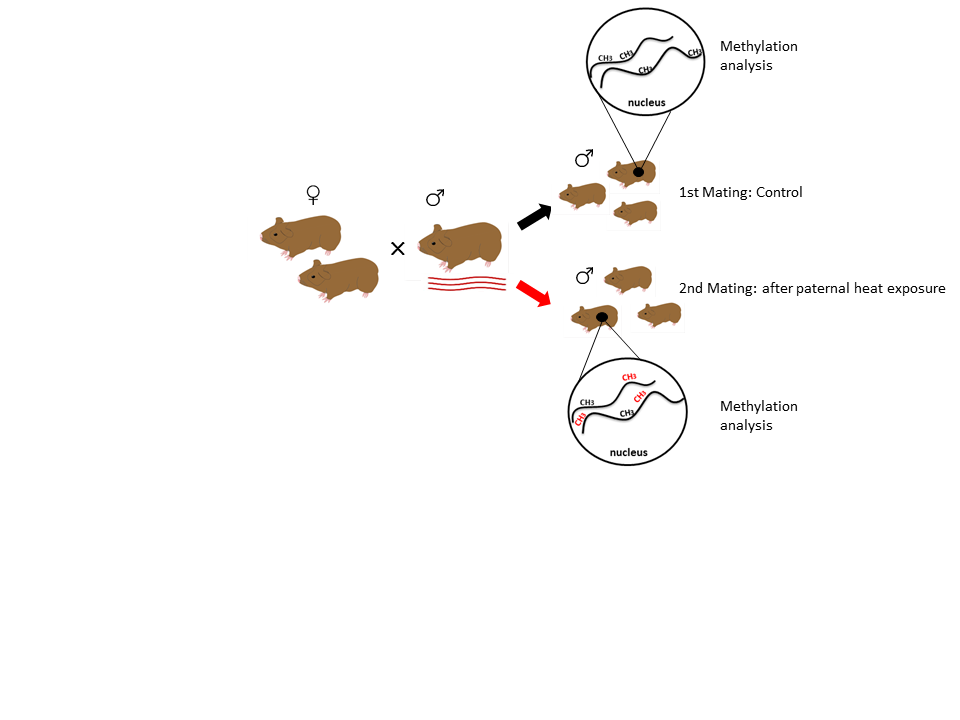
**

The experimental set-up shows that male Wild guinea pigs were exposure to increased temperature. They were mated to the same two female Wild guinea pigs and their sons were sampled before and after the exposure. Methylation patterns were analysed from DNA of whole livers of sons sired before (control: F1L_C_) and after heat exposure (F1L_H_). Potential changes in methylation of nuclear DNA are indicated by red CH_3_-groups.

**Table S1:** Methylation and expression levels of *Stat3*

| **Stat3 _scaffold53** | | | **Stat3 _ CpG loci _ methylation ratio** | | | | | | **Normalized Stat3 gene expression levels** |
| --- | --- | --- | --- | --- | --- | --- | --- | --- | --- |
| **No.** | **Animal ID** | **Treatment groups** | **CpG1_pos12161278** | **CpG2_pos12161335** | **CpG3_pos12161552** | **CpG4_pos12161581** | **CpG5_pos12166914** | **CpG6_pos12173162** |  |
| 1 | F1L_C_-F1 | control | 0.25 | 0.75 | 0.85 | 0.54 | 0.88 | 0 | 0.9528 |
| 2 | F1L_C_-F2 | control | NA | 0.75 | 0.7 | 0.36 | 0.86 | 0.67 | 0.5583 |
| 3 | F1L_C_-F3 | control | 0.13 | 0.43 | 0.89 | 0.6 | 0.5 | 0.36 | 0.5745 |
| 4 | F1L_C_-F4 | control | 0 | 0.42 | 0.6 | 0.3 | 0.63 | 0.18 | 1.0621 |
| 5 | F1L_C_-F5 | control | 0.33 | 0.73 | 0.73 | 0.63 | 0.5 | 0.42 | 0.6565 |
| 6 | F1L_C_-G1 | control | 0.13 | 0.14 | 0.7 | 0.76 | 0.43 | 0.21 | 0.4290 |
| 7 | F1L_C_-G2 | control | 0.11 | 0.55 | 0.87 | 0.87 | 0.83 | 0 | 0.3101 |
| 8 | F1L_C_-H1 | control | 0 | 0.5 | 0.7 | 0.79 | 0.55 | 0.36 | 0.6540 |
| 9 | F1L_C_-H2 | control | 0 | 0.48 | 0.44 | 0.67 | 0.67 | 0 | 0.4710 |
| 10 | F1L_C_-H3 | control | 0 | 0.6 | 0.56 | 0.25 | 0.75 | 0.25 | 0.6241 |
| 11 | F1L_C_-H4 | control | 0 | 0.25 | 0.71 | 0.57 | 0.75 | 0.13 | 0.4433 |
| 12 | F1L_C_-I1 | control | 0.17 | 0.17 | 0.76 | 0.92 | 1 | 0.25 | 0.5733 |
| 13 | F1L_C_-I2 | control | 0.09 | NA | NA | NA | 1 | 0.43 | 0.4666 |
| 14 | F1L_C_-J1 | control | 0.25 | 0.46 | 0.67 | 0.67 | 0 | 0.38 | 0.4936 |
| 15 | F1L_C_-J2 | control | 0.25 | 0.71 | 0.45 | 0.82 | 0.6 | 0.18 | 1.1916 |
| 16 | F1L_C_-J3 | control | 0 | 0.43 | 0.69 | 0.69 | 0.83 | 0 | 0.6981 |
| 17 | F1L_H_-F1 | heat | 0 | 0.67 | 1 | 1 | 1 | 0 | 0.4093 |
| 18 | F1L_H_-F2 | heat | 0.33 | 0.42 | 1 | 0.75 | 0.4 | 0.3 | 0.3175 |
| 19 | F1L_H_-G1 | heat | 0.25 | 0.5 | 1 | 0.8 | 1 | 0.38 | 0.2634 |
| 20 | F1L_H_-G2 | heat | 0.2 | 0.8 | 0.93 | 0.86 | 0.57 | 0.21 | 0.1777 |
| 21 | F1L_H_-G3 | heat | 0 | 0.6 | 0.63 | 0.67 | 0.57 | 0.25 | 0.2543 |
| 22 | F1L_H_-G4 | heat | 0 | 0.83 | 0.67 | 1 | 0.88 | 1 | 0.1632 |
| 23 | F1L_H_-H1 | heat | 0.17 | 0.47 | 0.67 | 0.33 | 1 | 0.36 | 0.3414 |
| 24 | F1L_H_-H2 | heat | 0 | 0.33 | 0.83 | 1 | 0.5 | 0.6 | 0.2866 |
| 25 | F1L_H_-H3 | heat | NA | 0.6 | 0.91 | 0.64 | 0.38 | 0.5 | 0.6115 |
| 26 | F1L_H_-H4 | heat | 0.14 | 0.6 | 1 | 0.8 | 0.44 | 0.45 | 0.1867 |
| 27 | F1L_H_-H5 | heat | 0.23 | 0.69 | 0.82 | 0.82 | 0.54 | 0.77 | 0.3425 |
| 28 | F1L_H_-I1 | heat | 0.13 | 0.53 | 0.92 | 0.8 | 0.4 | 0.25 | 0.1867 |
| 29 | F1L_H_-I2 | heat | 0.6 | 0.53 | 0.74 | 0.59 | 0 | 0.25 | 0.4478 |
| 30 | F1L_H_-I3 | heat | 0.2 | 0.6 | 0.67 | 0.67 | 0 | 0.4 | 0.1948 |
| 31 | F1L_H_-I4 | heat | 0.2 | 0.36 | 0.77 | 0.66 | 0.8 | 0.6 | 0.3842 |
| 32 | F1L_H_-I5 | heat | 0 | 0.5 | 0.71 | 1 | 0.43 | 0.33 | 1.4775 |
| 33 | F1L_H_-I6 | heat | 0 | 0.71 | 0.69 | 0.81 | 0.67 | 0.45 | 0.3622 |
| 34 | F1L_H_-J1 | heat | 0.11 | 0.71 | 0.8 | 0.87 | 0.25 | 0.12 | 0.3611 |

Wild guinea pig sons Stat3 methylation and expression levels are listed per individual. Liver samples were taken before and after the father`s heat exposure (F1L_C_; N=16 and F1L_H_; N= 18). Treatment groups are the sons (F_n_-J_n_) of fathers either untreated (control: F1L_C_-F1 - F1L_H_-J1) or exposed for 2 months to 30°C ambient temperature (heat: F1L_H_-F1 - F1L_H_-J1).
